# 1,5-Dimethyl-3-oxo-2-phenyl-2,3-di­hy­dro-1*H*-pyrazol-4-aminium 2-hydroxy­benzoate

**DOI:** 10.1107/S1600536809045760

**Published:** 2009-11-07

**Authors:** A. Chitradevi, S. Athimoolam, B. Sridhar, S. Asath Bahadur

**Affiliations:** a Department of Physics, Sri Subramanya College of Engineering & Technology, Palani 624 615, India; b Department of Physics, University College of Engineering, Anna University Tirunelveli, Nagercoil 629 004, India; c Laboratory of X-ray Crystallography, Indian Institute of Chemical Technology, Hyderabad 500 007, India; d Department of Physics, Kalasalingam University, Krishnan Koil 626 190, India

## Abstract

In the title salt, C_11_H_14_N_3_O^+^·C_7_H_5_O_3_^−^, the phenyl ring of the cation is oriented at an angle of 67.0 (1)° with respect to the five-membered pyrazolone ring. The carboxyl­ate plane of the anion is twisted out from the plane of the aromatic ring at an angle of 13.7 (3)°. In the crystal, the cations form hydrogen-bonded dimers with an *R*_2_^2^(10) ring motif. The salicylate anion has an intra­molecular O—H⋯O hydrogen bond.

## Related literature

For the biological and pharmacological importance of pyrazolone derivatives and 4-amino­anti­pyrene compounds, see: Filho *et al.* (1998 ▶); Jain *et al.* (2003 ▶); Mishra (1999 ▶); Sondhi *et al.* (1999 ▶); Sondhi *et al.* (2001 ▶). For similar hydrogen-bonded structures, see: Athimoolam & Natarajan (2006*a* ▶,*b* ▶,*c* ▶); Athimoolam & Rajaram (2005 ▶). For hydrogen bonding inter­actions and graph-set notations, see: Desiraju (1989 ▶); Etter *et al.* (1990 ▶). For a description of the Cambridge Structural Database, see: Allen (2002 ▶). 
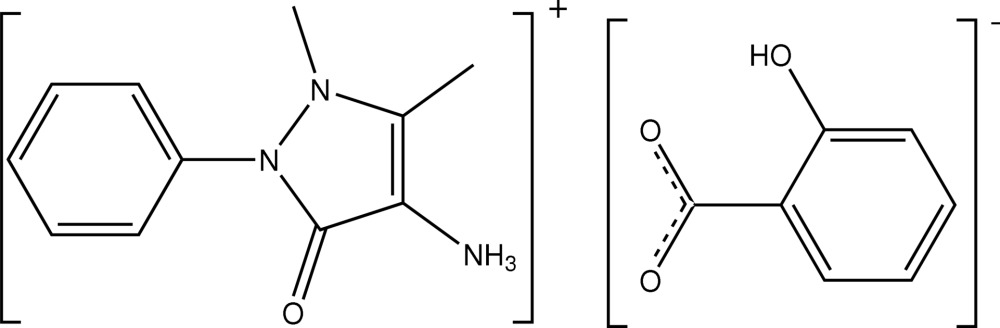


## Experimental

### 

#### Crystal data

C_11_H_14_N_3_O^+^·C_7_H_5_O_3_^−^
*M* *_r_* = 341.36
Monoclinic, 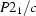

*a* = 8.3182 (6) Å
*b* = 23.3006 (16) Å
*c* = 8.8503 (6) Å
β = 101.517 (1)°
*V* = 1680.8 (2) Å^3^
*Z* = 4
Mo *K*α radiation
μ = 0.10 mm^−1^
*T* = 293 K
0.24 × 0.13 × 0.12 mm

#### Data collection

Bruker SMART APEX CCD area-detector diffractometer
Absorption correction: none
16025 measured reflections
2959 independent reflections
2599 reflections with *I* > 2σ(*I*)
*R*_int_ = 0.020

#### Refinement

*R*[*F* ^2^ > 2σ(*F* ^2^)] = 0.041
*wR*(*F* ^2^) = 0.113
*S* = 1.05
2959 reflections
229 parameters
H-atom parameters constrained
Δρ_max_ = 0.18 e Å^−3^
Δρ_min_ = −0.18 e Å^−3^

### 

Data collection: *SMART* (Bruker, 2001 ▶); cell refinement: *SAINT* (Bruker, 2001 ▶); data reduction: *SAINT*; program(s) used to solve structure: *SHELXTL/PC* (Sheldrick, 2008 ▶); program(s) used to refine structure: *SHELXTL/PC*; molecular graphics: *Mercury* (Macrae *et al.*, 2006 ▶ ) and *PLATON* (Spek, 2009 ▶); software used to prepare material for publication: *SHELXTL/PC*.

## Supplementary Material

Crystal structure: contains datablocks global, I. DOI: 10.1107/S1600536809045760/bt5124sup1.cif

Structure factors: contains datablocks I. DOI: 10.1107/S1600536809045760/bt5124Isup2.hkl

Additional supplementary materials:  crystallographic information; 3D view; checkCIF report

## Figures and Tables

**Table 1:** Hydrogen-bond geometry (Å, °)

*D*—H⋯*A*	*D*—H	H⋯*A*	*D*⋯*A*	*D*—H⋯*A*
N5—H5*A*⋯O4^i^	0.89	1.88	2.696 (2)	151
N5—H5*B*⋯O41	0.89	2.20	2.949 (2)	142
N5—H5*B*⋯O42	0.89	2.17	2.972 (2)	150
N5—H5*C*⋯O41^ii^	0.89	1.82	2.705 (2)	175
O43—H43*A*⋯O42	0.82	1.79	2.524 (3)	148

Symmetry codes: (i) 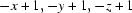; (ii) 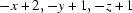.

**Fig. 1.**
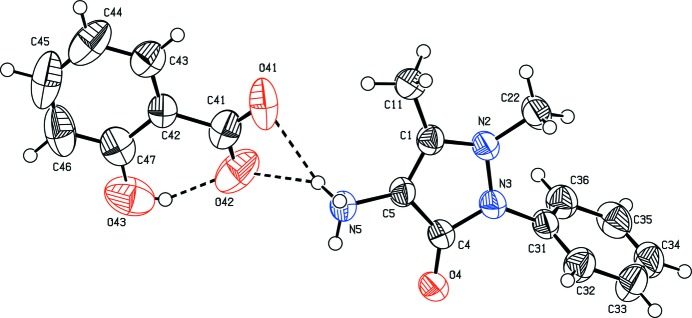
The molecular structure of the title compound with atom numbering scheme and 50% probability displacement ellipsoids. H-bonds are shown as dashed lines.

**Fig. 2.**
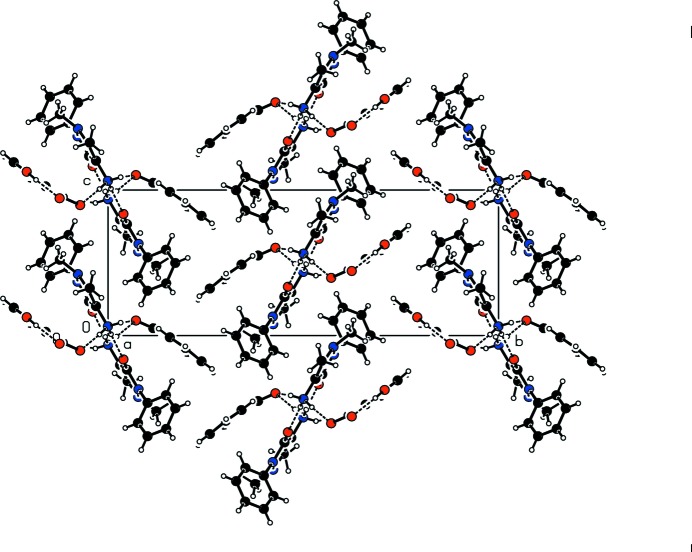
Packing diagram of the title compound viewed down the a-axis. H-bonds are shown as dashed lines.

**Fig. 3.**
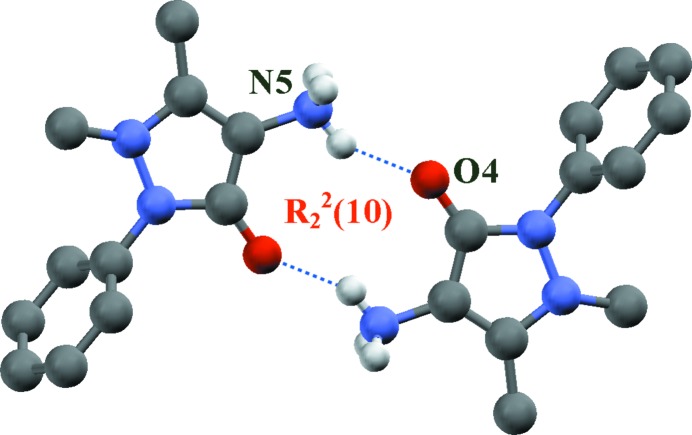
Inversion related ring R22(10) motif. Hydrogen atoms not involved in hydrogen bonds (dashed lines) are omitted for clarity.

**Fig. 4.**
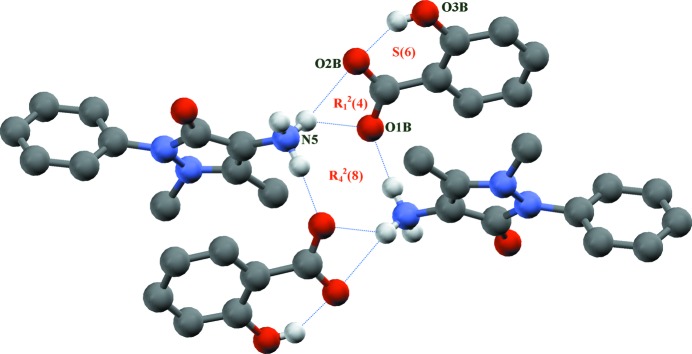
Intermolecular ring R12(4) and R42(8) motifs and intramolecular S(6) motif. Hydrogen atoms not involved in hydrogen bonds (dashed lines) are omitted for clarity.

## supplementary crystallographic information

### Comment

4-Aminoantipyrene, which contain pyrazolone ring, is an important compound in the class analgesic agent in otic solutions in combination with other analgesic such as benzocaine and phenylephrine. Pyrazolone is a five-membered lactam ring compound containing two N atoms and ketone in the same molecule. Lactam structure is an active nucleaus in pharmacological activity, especially in the class of nonsteroidal antiinflammatory agents used in the treatment of arthritis and other musculo skeletal and joint disorders. Pyrazolone derivatives, as lactam structure related compounds, are also widely used in preparing dyes and pigments. 4-aminoantipyrene and its derivatives have potential biological activities (Jain *et al.*, 2003). Analgesic and antiinflammatory activities of the 4-aminoantipyrene complexes were extensively studied and reported (Filho *et al.*, 1998; Sondhi *et al.*, 1999). Apart from that, antimicrobial and anticancer activity of the 4-aminoantipyrine derivatives and their metal complexes caught the attention of many researchers during last decade (Mishra, 1999; Sondhi *et al.*, 2001). As intra- and intermolecular hydrogen bonding interactions play a key role in bio-molecular interactions we are interested on the structural elucidation of potentially bioactive compounds and their hydrogen bonding interactions in different environments. Thus, we are concerned with the biomolecular hydrogen bonding interactions through their X-ray analyses of crystalline complexes involving drugs and vitamins with inorganic and organic acids (Athimoolam & Rajaram, 2005; Athimoolam & Natarajan, 2006*a*-c).

Intermolecular forces also play very essential role in the formation of supramolecular organic systems. The phenomenon of hydrogen bonding enlightens the area of molecular recognition, crystal-engineering research and organic synthons for supramolecular research (Desiraju, 1989). Carboxylic acids and amines are two commonly used functional groups in crystal engineering because they generally form robust architectures *via *O—H···O and N—H···O hydrogen-bonded interactions (Etter *et al.*, 1990). 4-aminoantipyrene is one of the such important ligands since it has potential sites for hydrogen bonding interactions, *viz*., the amine N atom (as donor) and carbonyl O atom (as acceptor). Consideration of these above specifics and to study the supramolecular geometry through hydrogen bonding extensions, the present investigation was undertaken. 4-aminoantipyrene was treated with salicylic acid and the title compound is crystallized.

The asymmetric unit of (I) consists of one single charged protonated 4-aminoantipyrene cation and a deprotonated salicylate anion (Fig 1). Interatomic distances and angles are normal and in good agreement with the similar structures (Allen, 2002). The expected proton transfer from salicylic acid to 4-aminoantipyrene is established at N5 atom. The protonation on the N site of the cation is evidenced from the elongated C—N bond distance and the deprotonation on anion is confirmed from the COO^-^ symmetric bond distances (Table 1). The phenyl ring of the cation is oriented with an angle of 67.0 (1)° to the five membered pyrazolone ring. Also, in the asymmeric unit, the phenyl ring of the cation is making a dihedral angle of 87.5 (1)° with the phenyl ring of the anion. The carboxylate plane of the salicylate anion is twisted from the plane of the aromatic ring with an angle of 13.7 (3)°. The twisting of carboxylate plane can be associated with the hydrogen bonding interactions of amino group of the cation. Due to the packing specificity of the crystal, one of the methyl atoms (C22) of the cation is slightly out of plane of the five-membered pyrzalone ring with the distance of 0.542 (3) Å.

The most elegant aspect of the present work is found not only in the molecular structure but also in the crystal packing *via* N—H···O and O—H···O hydrogen bonds. Fig. 2 shows the aggregation of the molecules around the inversion centres of the unit cell through ring motifs. As a characterestic H-bond, salicylate anion consists a self associated intramolecular S(6) motif through O—H···O hydrogen bond. The amino group of the cation is involved in two two-centered and one three-centered hydrogen bonds. The amino and carbonyl O atom of the cation is involving in N—H···O hydrogen bond which leads to a classical molecular dimerization through the ring *R*_2_^2^(10) motif around the inversion center of the unit cell (Fig. 3). Other two H atoms of amino group are involved in N—H···O hydrogen bonds with the adjascent salicylate anions. This leads to another ring *R*_4_^2^(8) motif formed through two cations and two anions (Fig. 4). This ring motif is further accompanied with another adajascent ring *R*_1_^2^(4) motif through the bifuracted (two-centered) hydrogen bond. These three intermolecular ring motifs are intersected and extending along the *a* axis of the unit cell. This leads to hydrophilic region at the plane *y*=1/2 which are sanwitched between the hydrophobic regions at *y*=1/4 and 3/4.

### Experimental

The title compound was crystallized from the aqueous mixtures of 4-aminoantipyrene with salicylic acid, in the stochiometric ratio of 1:1 at room temperature by the technique of slow evaporation.

### Refinement

All the H atoms were positioned geometrically and refined using a riding model, with C—H = 0.93 Å (aromatic) & 0.96 Å (methyl), N—H = 0.86 Å and O—H=0.82 Å and *U*_iso_(H) = 1.2–1.5 *U*_eq_ (parent atom).

### Crystal data

**Table:**

C_11_H_14_N_3_O^+^·C_7_H_5_O_3_^−^	*F*(000) = 720
*M**_r_* = 341.36	*D*_x_ = 1.349 Mg m^−^^3^*D*_m_ = 1.339 Mg m^−^^3^*D*_m_ measured by flotation technique using a liquid-mixture of xylene and carbon tetrachloride
Monoclinic, *P*2_1_/*c*	Mo *K*α radiation, λ = 0.71073 Å
Hall symbol: -P 2ybc	Cell parameters from 3884 reflections
*a* = 8.3182 (6) Å	θ = 2.6–23.9°
*b* = 23.3006 (16) Å	µ = 0.10 mm^−^^1^
*c* = 8.8503 (6) Å	*T* = 293 K
β = 101.517 (1)°	Needle, light orange
*V* = 1680.8 (2) Å^3^	0.24 × 0.13 × 0.12 mm
*Z* = 4	

### Data collection

**Table:**

Bruker SMART APEX CCD area-detector diffractometer	2599 reflections with *I* > 2σ(*I*)
Radiation source: fine-focus sealed tube	*R*_int_ = 0.020
graphite	θ_max_ = 25.0°, θ_min_ = 1.8°
ω scans	*h* = −9→9
16025 measured reflections	*k* = −27→27
2959 independent reflections	*l* = −10→10

### Refinement

**Table:**

Refinement on *F*^2^	Primary atom site location: structure-invariant direct methods
Least-squares matrix: full	Secondary atom site location: difference Fourier map
*R*[*F*^2^ > 2σ(*F*^2^)] = 0.041	Hydrogen site location: inferred from neighbouring sites
*wR*(*F*^2^) = 0.113	H-atom parameters constrained
*S* = 1.05	*w* = 1/[σ^2^(*F*_o_^2^) + (0.0515*P*)^2^ + 0.4777*P*] where *P* = (*F*_o_^2^ + 2*F*_c_^2^)/3
2959 reflections	(Δ/σ)_max_ < 0.001
229 parameters	Δρ_max_ = 0.18 e Å^−^^3^
0 restraints	Δρ_min_ = −0.18 e Å^−^^3^

### Special details

**Table:**

Geometry. All e.s.d.'s (except the e.s.d. in the dihedral angle between two l.s. planes) are estimated using the full covariance matrix. The cell e.s.d.'s are taken into account individually in the estimation of e.s.d.'s in distances, angles and torsion angles; correlations between e.s.d.'s in cell parameters are only used when they are defined by crystal symmetry. An approximate (isotropic) treatment of cell e.s.d.'s is used for estimating e.s.d.'s involving l.s. planes.
Refinement. Refinement of *F*^2^ against ALL reflections. The weighted *R*-factor *wR* and goodness of fit *S* are based on *F*^2^, conventional *R*-factors *R* are based on *F*, with *F* set to zero for negative *F*^2^. The threshold expression of *F*^2^ > σ(*F*^2^) is used only for calculating *R*-factors(gt) *etc*. and is not relevant to the choice of reflections for refinement. *R*-factors based on *F*^2^ are statistically about twice as large as those based on *F*, and *R*- factors based on ALL data will be even larger.

### Fractional atomic coordinates and isotropic or equivalent isotropic displacement parameters (Å^2^)

**Table:**

	*x*	*y*	*z*	*U*_iso_*/*U*_eq_	
C1	0.88415 (19)	0.55024 (6)	0.81347 (17)	0.0448 (3)	
C11	1.0648 (2)	0.54675 (8)	0.8259 (2)	0.0584 (4)	
H11A	1.0898	0.5168	0.7597	0.088*	
H11C	1.1164	0.5385	0.9306	0.088*	
H11B	1.1048	0.5827	0.7955	0.088*	
N2	0.81742 (15)	0.57725 (6)	0.92231 (15)	0.0484 (3)	
C22	0.8940 (2)	0.62350 (8)	1.0208 (2)	0.0625 (5)	
H22A	1.0071	0.6143	1.0603	0.094*	
H22B	0.8383	0.6285	1.1050	0.094*	
H22C	0.8874	0.6584	0.9621	0.094*	
N3	0.64810 (15)	0.57811 (6)	0.86977 (15)	0.0496 (3)	
C4	0.60726 (19)	0.54839 (7)	0.73428 (17)	0.0479 (4)	
O4	0.46461 (14)	0.53935 (6)	0.66452 (14)	0.0662 (4)	
C5	0.75999 (18)	0.53088 (6)	0.70220 (16)	0.0442 (3)	
N5	0.77414 (16)	0.50034 (6)	0.56334 (14)	0.0508 (3)	
H5A	0.6744	0.4926	0.5091	0.076*	
H5B	0.8286	0.4677	0.5883	0.076*	
H5C	0.8281	0.5219	0.5071	0.076*	
C31	0.54080 (18)	0.60115 (7)	0.96225 (17)	0.0471 (4)	
C32	0.4479 (2)	0.64820 (8)	0.9101 (2)	0.0619 (5)	
H32	0.4516	0.6640	0.8144	0.074*	
C33	0.3485 (2)	0.67208 (9)	1.0009 (3)	0.0743 (6)	
H33	0.2847	0.7040	0.9661	0.089*	
C34	0.3436 (2)	0.64892 (10)	1.1417 (2)	0.0697 (6)	
H34	0.2785	0.6657	1.2037	0.084*	
C35	0.4341 (2)	0.60110 (10)	1.1917 (2)	0.0680 (5)	
H35	0.4285	0.5850	1.2867	0.082*	
C36	0.5338 (2)	0.57665 (8)	1.10183 (19)	0.0588 (4)	
H36	0.5952	0.5441	1.1354	0.071*	
C41	0.9974 (3)	0.38422 (8)	0.5503 (2)	0.0628 (5)	
C42	1.0840 (2)	0.34016 (6)	0.47447 (17)	0.0493 (4)	
C43	1.2516 (2)	0.34179 (8)	0.4878 (2)	0.0653 (5)	
H43	1.3123	0.3703	0.5472	0.078*	
C44	1.3309 (3)	0.30161 (12)	0.4140 (3)	0.0940 (8)	
H44	1.4445	0.3022	0.4255	0.113*	
C45	1.2386 (5)	0.26065 (11)	0.3230 (3)	0.1051 (10)	
H45	1.2907	0.2340	0.2708	0.126*	
C46	1.0744 (4)	0.25848 (9)	0.3083 (3)	0.0926 (8)	
H46	1.0143	0.2309	0.2452	0.111*	
C47	0.9954 (3)	0.29676 (8)	0.3861 (2)	0.0652 (5)	
O41	1.0717 (2)	0.42857 (6)	0.60326 (16)	0.0872 (5)	
O42	0.8508 (2)	0.37591 (8)	0.5565 (2)	0.0980 (5)	
O43	0.8319 (2)	0.29178 (8)	0.3741 (2)	0.1032 (6)	
H43A	0.8004	0.3156	0.4299	0.155*	

### Atomic displacement parameters (Å^2^)

**Table:**

	*U*^11^	*U*^22^	*U*^33^	*U*^12^	*U*^13^	*U*^23^
C1	0.0488 (8)	0.0453 (8)	0.0415 (8)	0.0025 (6)	0.0118 (6)	−0.0012 (6)
C11	0.0492 (9)	0.0706 (11)	0.0555 (10)	0.0023 (8)	0.0105 (8)	−0.0107 (8)
N2	0.0448 (7)	0.0555 (8)	0.0444 (7)	−0.0020 (6)	0.0080 (5)	−0.0119 (6)
C22	0.0581 (10)	0.0703 (11)	0.0590 (10)	−0.0100 (8)	0.0115 (8)	−0.0230 (9)
N3	0.0436 (7)	0.0609 (8)	0.0443 (7)	0.0016 (6)	0.0088 (5)	−0.0139 (6)
C4	0.0489 (9)	0.0531 (9)	0.0406 (8)	0.0024 (7)	0.0066 (7)	−0.0087 (7)
O4	0.0479 (7)	0.0914 (9)	0.0559 (7)	0.0057 (6)	0.0025 (5)	−0.0288 (6)
C5	0.0494 (9)	0.0454 (8)	0.0383 (7)	0.0036 (6)	0.0098 (6)	−0.0049 (6)
N5	0.0518 (7)	0.0569 (8)	0.0437 (7)	0.0071 (6)	0.0095 (6)	−0.0109 (6)
C31	0.0440 (8)	0.0532 (9)	0.0450 (8)	−0.0031 (7)	0.0109 (6)	−0.0134 (7)
C32	0.0641 (11)	0.0649 (11)	0.0595 (10)	0.0077 (9)	0.0191 (8)	−0.0014 (8)
C33	0.0668 (12)	0.0720 (12)	0.0878 (15)	0.0153 (10)	0.0244 (11)	−0.0116 (11)
C34	0.0518 (10)	0.0900 (14)	0.0725 (12)	−0.0073 (10)	0.0244 (9)	−0.0326 (11)
C35	0.0634 (11)	0.0938 (15)	0.0514 (10)	−0.0145 (11)	0.0225 (9)	−0.0117 (10)
C36	0.0611 (10)	0.0660 (11)	0.0508 (9)	−0.0003 (8)	0.0149 (8)	−0.0047 (8)
C41	0.0865 (14)	0.0587 (11)	0.0490 (9)	0.0174 (10)	0.0277 (9)	0.0136 (8)
C42	0.0662 (10)	0.0418 (8)	0.0419 (8)	0.0044 (7)	0.0153 (7)	0.0065 (6)
C43	0.0689 (12)	0.0627 (11)	0.0668 (11)	0.0065 (9)	0.0199 (9)	0.0089 (9)
C44	0.0935 (17)	0.0945 (17)	0.1083 (19)	0.0351 (14)	0.0544 (15)	0.0320 (15)
C45	0.181 (3)	0.0629 (14)	0.0896 (18)	0.0378 (18)	0.071 (2)	0.0077 (13)
C46	0.165 (3)	0.0485 (11)	0.0661 (13)	0.0005 (14)	0.0285 (15)	−0.0055 (9)
C47	0.0919 (14)	0.0491 (10)	0.0537 (10)	−0.0046 (9)	0.0125 (9)	0.0113 (8)
O41	0.1512 (15)	0.0554 (8)	0.0651 (9)	0.0028 (8)	0.0456 (9)	−0.0106 (7)
O42	0.0848 (11)	0.1136 (13)	0.1090 (13)	0.0311 (9)	0.0514 (10)	0.0239 (10)
O43	0.0926 (12)	0.0962 (13)	0.1114 (14)	−0.0326 (9)	−0.0020 (10)	0.0149 (10)

### Geometric parameters (Å, °)

**Table:**

C1—C5	1.354 (2)	C33—C34	1.366 (3)
C1—N2	1.3585 (19)	C33—H33	0.9300
C1—C11	1.487 (2)	C34—C35	1.368 (3)
C11—H11A	0.9600	C34—H34	0.9300
C11—H11C	0.9600	C35—C36	1.381 (3)
C11—H11B	0.9600	C35—H35	0.9300
N2—N3	1.3924 (18)	C36—H36	0.9300
N2—C22	1.451 (2)	C41—O42	1.246 (2)
C22—H22A	0.9600	C41—O41	1.247 (2)
C22—H22B	0.9600	C41—C42	1.489 (2)
C22—H22C	0.9600	C42—C43	1.376 (3)
N3—C4	1.3675 (19)	C42—C47	1.395 (2)
N3—C31	1.4306 (19)	C43—C44	1.382 (3)
C4—O4	1.2414 (19)	C43—H43	0.9300
C4—C5	1.416 (2)	C44—C45	1.379 (4)
C5—N5	1.4450 (18)	C44—H44	0.9300
N5—H5A	0.8900	C45—C46	1.347 (4)
N5—H5B	0.8900	C45—H45	0.9300
N5—H5C	0.8900	C46—C47	1.372 (3)
C31—C32	1.367 (2)	C46—H46	0.9300
C31—C36	1.373 (2)	C47—O43	1.348 (3)
C32—C33	1.380 (3)	O43—H43A	0.8200
C32—H32	0.9300		
			
C5—C1—N2	108.04 (13)	C31—C32—H32	120.3
C5—C1—C11	130.42 (14)	C33—C32—H32	120.3
N2—C1—C11	121.52 (13)	C34—C33—C32	120.15 (19)
C1—C11—H11A	109.5	C34—C33—H33	119.9
C1—C11—H11C	109.5	C32—C33—H33	119.9
H11A—C11—H11C	109.5	C33—C34—C35	120.15 (17)
C1—C11—H11B	109.5	C33—C34—H34	119.9
H11A—C11—H11B	109.5	C35—C34—H34	119.9
H11C—C11—H11B	109.5	C34—C35—C36	120.29 (18)
C1—N2—N3	107.41 (12)	C34—C35—H35	119.9
C1—N2—C22	125.26 (14)	C36—C35—H35	119.9
N3—N2—C22	118.91 (13)	C31—C36—C35	119.02 (18)
N2—C22—H22A	109.5	C31—C36—H36	120.5
N2—C22—H22B	109.5	C35—C36—H36	120.5
H22A—C22—H22B	109.5	O42—C41—O41	121.88 (19)
N2—C22—H22C	109.5	O42—C41—C42	118.59 (19)
H22A—C22—H22C	109.5	O41—C41—C42	119.52 (19)
H22B—C22—H22C	109.5	C43—C42—C47	118.73 (17)
C4—N3—N2	110.07 (12)	C43—C42—C41	121.05 (17)
C4—N3—C31	128.14 (13)	C47—C42—C41	120.21 (18)
N2—N3—C31	121.29 (12)	C42—C43—C44	120.8 (2)
O4—C4—N3	124.58 (14)	C42—C43—H43	119.6
O4—C4—C5	131.15 (14)	C44—C43—H43	119.6
N3—C4—C5	104.24 (13)	C45—C44—C43	118.8 (2)
C1—C5—C4	110.04 (13)	C45—C44—H44	120.6
C1—C5—N5	127.06 (14)	C43—C44—H44	120.6
C4—C5—N5	122.76 (13)	C46—C45—C44	121.2 (2)
C5—N5—H5A	109.5	C46—C45—H45	119.4
C5—N5—H5B	109.5	C44—C45—H45	119.4
H5A—N5—H5B	109.5	C45—C46—C47	120.4 (2)
C5—N5—H5C	109.5	C45—C46—H46	119.8
H5A—N5—H5C	109.5	C47—C46—H46	119.8
H5B—N5—H5C	109.5	O43—C47—C46	118.8 (2)
C32—C31—C36	120.94 (15)	O43—C47—C42	121.22 (18)
C32—C31—N3	118.89 (15)	C46—C47—C42	120.0 (2)
C36—C31—N3	120.16 (15)	C47—O43—H43A	109.5
C31—C32—C33	119.41 (18)		
			
C5—C1—N2—N3	−4.57 (17)	C36—C31—C32—C33	1.5 (3)
C11—C1—N2—N3	174.07 (14)	N3—C31—C32—C33	−177.62 (17)
C5—C1—N2—C22	−151.94 (16)	C31—C32—C33—C34	0.2 (3)
C11—C1—N2—C22	26.7 (2)	C32—C33—C34—C35	−1.6 (3)
C1—N2—N3—C4	4.24 (17)	C33—C34—C35—C36	1.4 (3)
C22—N2—N3—C4	154.04 (15)	C32—C31—C36—C35	−1.7 (3)
C1—N2—N3—C31	176.75 (14)	N3—C31—C36—C35	177.43 (15)
C22—N2—N3—C31	−33.5 (2)	C34—C35—C36—C31	0.2 (3)
N2—N3—C4—O4	176.11 (16)	O42—C41—C42—C43	−168.40 (17)
C31—N3—C4—O4	4.2 (3)	O41—C41—C42—C43	12.8 (2)
N2—N3—C4—C5	−2.15 (17)	O42—C41—C42—C47	12.6 (2)
C31—N3—C4—C5	−174.00 (15)	O41—C41—C42—C47	−166.22 (16)
N2—C1—C5—C4	3.34 (18)	C47—C42—C43—C44	0.6 (3)
C11—C1—C5—C4	−175.14 (16)	C41—C42—C43—C44	−178.37 (17)
N2—C1—C5—N5	179.12 (14)	C42—C43—C44—C45	1.8 (3)
C11—C1—C5—N5	0.6 (3)	C43—C44—C45—C46	−1.6 (4)
O4—C4—C5—C1	−178.80 (18)	C44—C45—C46—C47	−1.0 (4)
N3—C4—C5—C1	−0.71 (18)	C45—C46—C47—O43	−177.1 (2)
O4—C4—C5—N5	5.2 (3)	C45—C46—C47—C42	3.4 (3)
N3—C4—C5—N5	−176.71 (14)	C43—C42—C47—O43	177.36 (17)
C4—N3—C31—C32	−72.0 (2)	C41—C42—C47—O43	−3.6 (2)
N2—N3—C31—C32	116.97 (17)	C43—C42—C47—C46	−3.2 (3)
C4—N3—C31—C36	108.9 (2)	C41—C42—C47—C46	175.77 (16)
N2—N3—C31—C36	−62.1 (2)		

### Hydrogen-bond geometry (Å, °)

**Table:**

*D*—H···*A*	*D*—H	H···*A*	*D*···*A*	*D*—H···*A*
N5—H5A···O4^i^	0.89	1.88	2.696 (2)	151
N5—H5B···O41	0.89	2.20	2.949 (2)	142
N5—H5B···O42	0.89	2.17	2.972 (2)	150
N5—H5C···O41^ii^	0.89	1.82	2.705 (2)	175
O43—H43A···O42	0.82	1.79	2.524 (3)	148

Symmetry codes: (i) −*x*+1, −*y*+1, −*z*+1; (ii) −*x*+2, −*y*+1, −*z*+1.
